# Psychological impact of motor impairment in tow forms of congenital muscular dystrophy

**DOI:** 10.1192/j.eurpsy.2023.1539

**Published:** 2023-07-19

**Authors:** I. Boujelbene, M. Chaabane, M. Guirat, D. Ben Touhemi, N. Gharbi, M. Yousr, H. Kamoun, I. Ben Ayed

**Affiliations:** 1 Department of Medical Genetic; 2 Department of Child Psychiatry, Hedi Chaker Hospital, Sfax, Tunisia

## Abstract

**Introduction:**

Congenital muscular dystrophies (CMDs) represent a heterogeneous group of early-onset muscle disorders presenting primarily with hypotonia and delayed motor development. Several genes are known to be responsible for CMDs, including the *LAMA2* gene, involved in merosin-deficient type 1A (MDC1A), and the *FKRP* gene involved in muscular dystrophy-dystroglycanopathy type B5 (MDDGB5). These two forms of CMD are autosomal recessive and are each characterized by the presence of a mutation with a founder effect in South Tunisia. Cognitive development associated with the founder mutation in the *LAMA2* gene (c.8007delT) is often conserved, whereas in the founder mutation of the *FKRP* gene (c.1364 C>A), motor impairment is associated with intellectual disability (ID).

**Objectives:**

To compare the psychological impact of motor impairment in children presenting these two forms of CMD and their families.

**Methods:**

The study consisted of a survey of parents of children with a confirmed diagnosis of MDC1A (5 from 3 unrelated families) or MDDGB5 (3 from 3 unrelated families). The correspondent founder mutation was already identified in the homozygous state by targeted sequencing. Participants’ parents completed the Parent Strengths and Difficulties Questionnaire (SDQ), a behavioral screening tool designed for children aged from 2 to 17 years. The SDQ assesses emotional symptoms, behavior problems, hyperactivity, and peer relationships; The SDQ Impact Supplement assesses the impact of all these children’s difficulties on their families.

**Results:**

The average age of the children was 4.95±3.92 with two children who were not assessable by the SDQ (age< 2 years). Unlike children with MDC1A, ID has been reported in all children with MDDGB5. The mean SDQ total score for children with MDC1A was 11, whereas the mean score for children with MDDGB5 was 14.875, reflecting greater difficulty for children with MDDGB5. The family impact score was higher in families with children with MDDGB5 than in children with MDC1A (10,5 vs 7), which may be due to the burden of management of the ID associated with the motor impairment. The more pronounced difficulties associated with MDDGB5 are likely to be related to the associated ID. Whereas in MDC1A, the difficulties observed are related to the direct impact of the motor impairment. The presence of cognitive disorders associated with a motor deficit aggravates behavioral adaptation and makes the management of these children more difficult.

**Conclusions:**

In the absence of a comparable study in the literature, the present is conducting future studies on the behavioral profile of children with CMD to obtain a better understanding of their difficulties in everyday life and to develop interventions adapted to their families

**Disclosure of Interest:**

None Declared

